# Recognition of refractory *Mycoplasma pneumoniae* pneumonia among *Myocoplasma pneumoniae* pneumonia in hospitalized children: development and validation of a predictive nomogram model

**DOI:** 10.1186/s12890-023-02684-1

**Published:** 2023-10-10

**Authors:** Meng Li, Xiang Wei, Shan-Shan Zhang, Shan Li, Su-Hong Chen, Su-Jie Shi, Shao-Hong Zhou, Da-Quan Sun, Qian-Ye Zhao, Yan Xu

**Affiliations:** 1 Pediatric Respiratory Ward I, Lianyungang Maternal and Child Health Care Hospital, No. 669, Qindongmen Street, Haizhou District, Lianyungang, 220003 Jiangsu China; 2 Medical College of Yangzhou University, Yangzhou, Jiangsu China; 3 Department of Pediatric Endocrinology, Lianyungang Maternal and Child Health Care Hospital, Lianyungang, Jiangsu China; 4 Neonatology Department, The Affiliated Hospital of Xuzhou Medical University, No. 99, Huaihai West Road, Quanshan District, Xuzhou, 221000 Jiangsu China

**Keywords:** Refractory *Mycoplasma pneumoniae* pneumonia, Nomogram, Receiver operating characteristic curve, Calibration curve, Decision curve analysis

## Abstract

**Backgroud:**

The current diagnostic criteria for refractory *Mycoplasma pneumoniae* pneumonia (RMPP) among *Mycoplasma pneumoniae* Pneumonia (MPP) are insufficient for early identification, and potentially delayed appropriate treatment. This study aimed to develop an effective individualized diagnostic prediction nomogram for pediatric RMPP.

**Methods:**

A total of 517 hospitalized children with MPP, including 131 with RMPP and 386 without RMPP (non-RMPP), treated at Lianyungang Maternal and Child Health Care Hospital from January 2018 to December 2021 were retrospectively enrolled as a development (modeling) cohort to construct an RMPP prediction nomogram. Additionally, 322 pediatric patients with MPP (64 with RMPP and 258 with non-RMPP, who were treated at the Affiliated Hospital of Xuzhou Medical University from June 2020 to May 2022 were retrospectively enrolled as a validation cohort to assess the prediction accuracy of model. Univariable and multivariable logistic regression analyses were used to identify RMPP risk factors among patients with MPP. Nomogram were generated based on these risk factors using the rms package of R, and the predictive performance was evaluated based on receiver operating characteristic (ROC) curves and using decision curve analysis (DCA).

**Results:**

Multivariate analysis revealed five significant independent predictors of RMPP among patients with MPP: age (hazard ratio [*HR*] 1.16, 95% confidence interval [*CI*] 1.08–1.33, *P* = 0.038), fever duration (*HR* 1.34, 95%*CI* 1.20–1.50, *P* < 0.001), lymphocyte count (*HR* 0.45, 95%*CI* 0.23–0.89, *P* = 0.021), serum D-dimer (D-d) level (*HR* 1.70, 95%*CI* 1.16–2.49, *P* = 0.006), and pulmonary imaging score (*HR* 5.16, 95%*CI* 2.38–11.21, *P* < 0.001). The area under the ROC curve was 90.7% for the development cohort and 96.36% for the validation cohort. The internal and external verification calibration curves were almost linear with slopes of 1, and the DCA curve revealed a net benefit with the final predictive nomogram.

**Conclusion:**

This study proposes a predictive nomogram only based on five variables. The nomogram can be used for early identification of RMPP among pediatric patients with MPP, thereby facilitating more timely and effective intervention.

**Supplementary Information:**

The online version contains supplementary material available at 10.1186/s12890-023-02684-1.

## Introduction

A nationwide *Mycoplasma pneumoniae pneumonia* (MPP) epidemic occurred in China in 2021, placing further pressure on hospitals already dealing with the worldwide coronavirus disease 2019 pandemic. Among patients with MPP, the incidence of refractory MPP (RMPP) is increasing [1, 2], RMPP can progress to necrotizing pneumonia, pulmonary embolism, plastic bronchitis, acute lung abscess, bronchiectasis, bronchiolitis obliterans, or unilateral hyperlucent lung during convalescence [3]. Thus, early recognition and appropriate management of RMPP are critical to avoid long-term pulmonary damage.

RMPP is diagnosed according to the clinical manifestations, such as fever, cough, wheezing and pulmonary changes on imaging, which do not improved, or are even aggravated after administration of appropriate macrolide antibiotic treatment for 7 days or longer [4, 5]. Macrolide-resistant *Mycoplasma pneumoniae* infection [6] and an excessively strong and sustained systemic inflammatory reaction [7] are the pathogenies of RMPP. Moreover, RMPP is challenging to diagnose in the early stages of MPP when application of immune modulators, and/or second-line antibiotic therapies, may be particularly effective, so most patients are likely to experience a period involving a sustained intrinsic immune response, which may result in irreversible intrapulmonary lesions [8].

Previous studies on potential predictors of RMPP have identified single risk factors, such as elevated serum L-lactate dehydrogenase (LDH) [9], D-dimer [10], interleukin (IL)-18 [11], and IL-17A [12], while others have identified combined predictors, such as high tumor necrosis factor-α expression, community-acquired respiratory distress syndrome toxin in bronchoalveolar lavage fluid [13], high serum C-reactive protein (CRP) level, high LDH level, high polymorphonuclear neutrophils (PMNs), and high D-dimer level combined with lung consolidation [14]. However, previous predictive methods utilizing these individual and combined factors have not been widely used in clinical practice because of a lack of quantitative scores for these indicators.

This study aimed to develop and validate a risk prediction nomogram model based on patients’ demographic characteristics, clinical manifestations, laboratory data and radiographic changes that can help in the early identification of RMPP among pediatric patients with MPP. This article is presented in accordance with the Transparent Reporting of a multivariate prediction model for Individual Prognosis Or Diagnosis checklist (Supplementary Material 1).

## Methods

### Populations and groups

This retrospective and observational cohort study was conducted at Lianyungang Maternal and Child Health Care Hospital, which is a 550-bed teaching hospital in Lianyungang, China, and at the Affiliated Hospital of Xuzhou Medical University, which is a 4500-bed teaching hospital in Xuzhou, China. The study was approved by the ethics committees of both hospitals and was conducted according to the tenets of the Declaration of Helsinki. The requirement for informed consent was waived due to the retrospective study design and anonymized patient data.

**Fig. 1:**
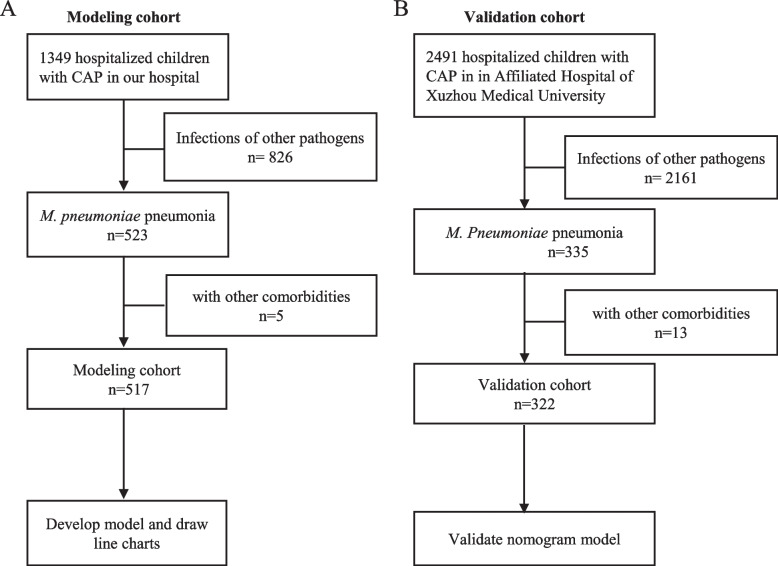
Screening flowchart of patients with *Mycoplasma. pneumoniae* pneumonia in the modeling cohort and validation cohorts

A total of 517 children with MPP who were treated from January 1, 2018, to December 31, 2021 at Lianyungang Maternal and Child Health Care Hospital and who were eligible for inclusion based on the inclusion/exclusion criteria were enrolled as a development (modeling) cohort. Additionally, 322 children with MPP who were treated at the Affiliated Hospital of Xuzhou Medical University from June 1, 2020, to May 31, 2022, were enrolled as a validation cohort to assess the predictive efficacy of the model. A flowchart of patient enrollment is shown in Fig. 1.

The inclusion criteria for RMPP were as follows: (a) hospitalized patients aged < 14 years, (b) diagnostic criteria for MPP according to the Chinese Expert Consensus on MPP [5], detailed in Supplementary Material 2, (c) macrolide-unresponsive MPP patients receiving intravenous azithromycin or erythromycin for 3 days, and a small dose of methylprednisolone (2-4 mg/kg.d), and (d) the clinical symptoms and radiological findings on chest X-ray or low-dose multislice spiral computed tomography scans did not improve or even aggravated [4], and (e) improvement and discharge from hospital after 7 to 14 days of treatment. Non-RMPP was defined as MPP showing fever relief, improvement in clinical signs, and obvious absorption according to chest imaging during the 3-day to 5-day azithromycin or erythromycin, and/or low-dose intravenous methylprednisolone treatment regimen. Patients with MPP coexisting with the following comorbidities were excluded: (a) immune dysfunction; (b) chronic pulmonary diseases, such as congenital ciliary dyskinesia, cystic fibrosis, bronchiectasis, diffuse interstitial lung disease, and bronchopulmonary dysplasia; and (c) congenital heart diseases.

Based on these criteria, patients with MPP in both the modeling and validation cohorts were divided into the RMPP and Non-RMPP groups (development cohort: 131 patients with RMPP and 386 patients with non-RMPP patients; validation cohort: 64 patients with RMPP and 258 patients with non-RMPP patients).

### Data collection

Baseline clinical and demographic variables were recorded on the first day of admission. These variables included age; fever duration (days); white blood cell (WBC) count; platelet (PLT) count; lymphocyte count; proportion of PMNs and lymphocytes; hemoglobin (Hb) level; serum levels of CRP; glutamic-oxaloacetic transaminase (GOT); glutamic-pyruvic transaminase (GPT); LDH; creatine kinase (CK); creatine kinase-MB (CK-MB) isoenzyme; and D-dimer; and pulmonary imaging score. Values for the above mentioned indictors were determined using an automatic blood analyzer (SYSMEX XS900i, Japan), automatic coagulation analyzer (SYSMEX CS-2000i, Japan), and automatic biochemical analyzer (HITACHI 7600–020, Japan) at Lianyungang Maternal and Child Health Care Hospital and the Affiliated Hospital of Xuzhou Medical University. Two clinical laboratories participated in inter-laboratory quality assessments in Jiangsu Province and China, which fully guaranteed the homogeneity of the experimental results.

### Chest imaging score

The worse radiological finding of consolidative lesions and pleural effusion in children with MPP were associated with more severe clinical course and poor treatment response [15, 16]. Severity scores were assigned for specific chest imaging signs described previously [17]. In order to incorporate chest imaging findings into regression analysis, we created the chest imaging score of MPP cases (Table 1). These scores were not cumulative; rather, the highest score was used to calculate the total chest imaging score. Pulmonary imaging signs were scored independently by two radiologists, and any differences were resolved by discussion. If consensus was not reached, disagreements were settled by a third radiologist.

**Table 1:** Chest imaging score

Chest imaging findings	Yes	No
Non-consolidation	1	0
Consolidation	2	0
Consolidation and a small amount of pleural effusion	3	0
Consolidation and a medium to large amount of pleural effusion	4	0

Non-consolidation: patchy infiltration, or localized reticulonodular infiltration, or parahilar peribronchial infiltration; Consolidation: lobar or segmental consolidation and/or atelectasis; A small amount of pleural efusion: the angle of the costal diaphragm becomes dull; A medium to large amount of pleural efusion: a large uniform dense shadow in the lower pleural cavity, the upper boundary is curved, the concave surface is upward, and the highest point is in the armpit; Even the mediastinum is pushed to the opposite side. The scores of various chest imaging changes were not cumulative, and the highest score was used as the imaging score

### Statistical analysis

Normally distributed data, as determined by the QQ plot method, are expressed as the mean ± standard deviation, while data with a skewed distribution on QQ plots are expressed as the median (interquartile range). Normally distributed data were compared using the independent-samples Student’s *t*-test, while skewed data were compared using the Mann–Whitney *U* test. Categorical variables were compared using Fisher’s exact test.

A prediction nomogram was constructed from all variables significantly associated with RMPP risk (*p* < 0.05) in the multivariate analysis using stepwise logistic regression. The survminer package of R software was used to draw forest plots, and the rms package of R was applied to transform the final regression model into a nomogram. The performance of the prediction model was evaluated to determine its discrimination ability by receiver operating characteristic (ROC) curve analysis, while calibration ability was evaluated by constructing calibration plots using 1000 bootstrap samples. The accuracy of the RMPP risk nomogram was assessed using decision curve analysis (DCA).

All statistical analyses were performed using SPSS 24.0 (IBM Corp, Armonk, NY, USA) and graphics-based R software packages (version 4.0.0 http:// www.r- project. org), including “foreign,” “rms,” “readxl,” “rmda,” “ResourceSelection,” “ROCR,” “forestplot,” and “forestmodel.” The use of R software is described in Supplementary Materials 3 and 4. A two-sided α of < 0.05 was considered statistically significant.

## Results

### General information

A total of 3,840 patients with community acquired pneumonia were screened within the study period, and 839 patients with MPP were enrolled in the development (modeling) and validation cohorts (131 patients with RMPP and 386 patients with non-RMPP in the modeling cohort; 64 patients with RMPP and 258 patients with non-RMPP cases in the validation cohort) (Fig. 1). The clinical characteristics of the two patient cohorts are compared in Table 2. There were no significant differences in baseline variables (age; fever duration; WBC count; PLT count; PMN and lymphocyte counts; proportions of PMNs and lymphocytes; serum levels of GOT, CK, and D-dimer; and chest imaging scores) between the modeling and validation cohorts (all *P* > 0.05).

**Table 2:** Admission characteristics of children with *Mycoplasma. pneumoniae* pneumonia in two groups according to their subsequent clinical outcome

Items	Modeling cohort (*n* = 517)	Validation cohort (*n* = 322)	*P*-value
Age (years)	5.42 (3.42 ~ 7.00)	5.08 (3.00 ~ 10.00)	0.171
Fever days	4.00 (1.00 ~ 6.00)	3.00 (3.00 ~ 6.00)	0.491
WBC (10^9^/L)	8.20 (6.35 ~ 10.84)	8.42 (6.34 ~ 11.49)	0.318
PMNs %	57.34 ± 15.32	58.00 ± 17.34	0.577
L %	33.16 ± 14.17	32.45 ± 15.89	0.514
PMNs (10^9^/L)	4.62 (3.26 ~ 6.61)	4.71 (3.22 ~ 7.22)	0.506
L (10^9^/L)	2.46 (1.73 ~ 3.58)	2.36 (1.74 ~ 3.50)	0.658
Hb (g/L)	129.26 ± 10.00	127.15 ± 12.58	0.007
PLT (10^9^/L)	302.84 ± 106.14	299.13 ± 101.78	0.617
CRP (ug/ml)	4.09 (1.02 ~ 9.97)	4.80 (1.36 ~ 14.59)	0.028
ALT (U/L)	15.50 (11.70 ~ 20.20)	14.30 (11.00 ~ 18.90)	0.009
AST (U/L)	31.40 (25.50 ~ 40.00)	32.90 (25.80 ~ 40.30)	0.237
LDH (U/L)	302.00 (259.40 ~ 379.50)	320.70 (272.00 ~ 427.00)	0.011
CK (U/L)	87.70 (57.40 ~ 133.60)	87.80 (59.00 ~ 132.00)	0.968
CK-MB (U/L)	23.00 (15.002 ~ 35.90)	18.50 (1.33 ~ 33.70)	< 0.001
D-d (mg/L)	0.43 (0.28 ~ 0.74)	0.41 (0.25 ~ 0.82)	0.307
Pumonary imaging score	542	352	0.734
1	416 (80.50%)	255(79.20%)	
2	50 (9.70%)	41(12.70%)	
3	19(3.70%)	4(1.20%)	
4	32(6.20%)	22(6.80%)	

Normal distribution data was presented as mean ± standard deviation. Skewness distribution data was presented as medians (one-quarter ~ three-quarters). *P* < 0.05 was considered statistically significant

*Abbreviations*: *WBC* white blood cells, *PMNs* polymorphonuclear neutrophils, *L* lymphocytes, *Hb* hemoglobin, *PLT* platelet, *CRP* C-reactive protein, *AST* glutamic oxaloacetic transaminase, *ALT* glutamic-pyruvic transaminase, *LDH* L-lactate dehydrogenase, *CK* creatine kinase, *CK-MB* creatine kinase-MB, *D-d* D-dimer

### Predictor selection of nomogram

**Fig. 2:**
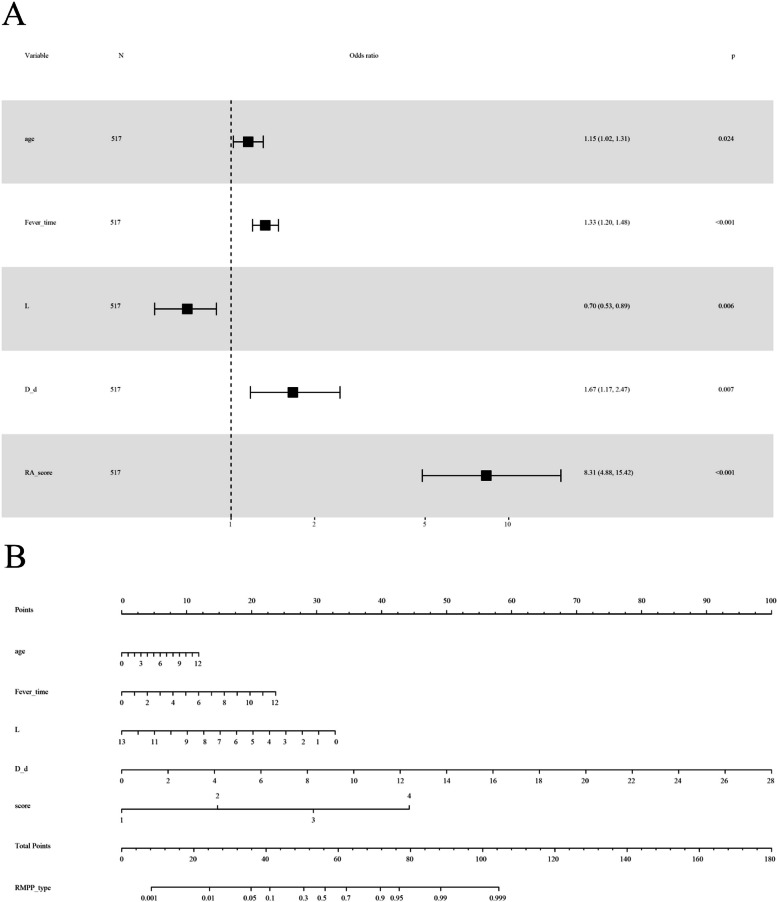
Forest chart and nomogram model. **A** Forest chart of the analysis of independent risk factors in the modeling cohort. **B** Nomogram model for early prediction of refractory *Mycoplasma pneumoniae* pneumonia. Instructions for using the nomogram: (a) Draw a line perpendicular from each of the five variables to the top line labeled “Points” to obtain the corresponding number of points; (b) add the points obtained from each of the five variables to obtain the total number of points; and (c) draw a line descending from the axis labeled “Total points” until it intercepts the RMPP type. The risk value corresponding to the RMPP type represents the specific risk at which RMPP will occur

In the modeling cohort, age, fever duration (in days), WBC count, lymphocyte count, PLT count, proportions of PMNs and lymphocytes, and chest imaging scores, as well as serum CRP, GPT, LDH, CK-MB, and D-dimer levels were significantly higher among the patients with RMPP than among the patients with non-RMPP patients (*P* < 0.05) (Table 3). All of the above mentioned changes were also observed in the validation cohort, with the exception of WBC count and serum GPT level, implying that these two indicators were not significantly different between RMPP and Non-RMPP group in the validation cohort (Table 4). The multivariate regression analysis identified age (hazard ratio [*HR*] 1.16 for every 1-year increase, 95% confidence interval [*CI*] 1.08–1.33, *P* < 0.05), fever duration (*HR* 1.34 for every 1-day increase, 95% *CI* 1.20–1.50, *P* < 0.001), serum D-dimer level (*HR* 1.70 for every 1 mg/L increase, 95% *CI* 1.16–2.49, *P* < 0.05), and pulmonary imaging score (*HR* 5.16 for every 1-point increase, 95% *CI* 2.38–11.21, *P* < 0.001) as independent risk factors for RMPP in the modeling cohort (Table 5). Of these factors, imaging score had the greatest influence on RMPP risk, while lymphocyte count (*HR* 0.45 for every 1 × 10^9^/L increase, 95% *CI* 0.23–0.89, *P* < 0.05) was a protective factor (Fig. 2A).

**Table 3:** Admission characteristics of children with *Mycoplasma pneumoniae * pneumonia in the modeling cohort

Items	Non-RMPP (*n* = 386)	RMPP (*n* = 131)	*P*-value
Age (years)	4.75(3.00 ~ 6.00)	6.00 (5.16 ~ 7.00)	< 0.001
Fever days	3.00 (1.00 ~ 5.00)	6.00 (4.00 ~ 7.00)	< 0.001
WBC (10^9^/L)	8.35 (6.58 ~ 11.19)	7.48 (5.84 ~ 10.13)	0.022
PMNs %	55.17 ± 15.34	63.72 ± 13.41	< 0.001
L %	35.39 ± 14.10	26.58 ± 12.26	< 0.001
PMNs (10^9^/L)	4.62 (3.26 ~ 6.61)	4.71 (3.22 ~ 7.22)	0.506
L (109/L)	2.46 (1.73 ~ 3.58)	2.36 (1.74 ~ 3.50)	< 0.001
Hb (g/L)	129.11 ± 9.91	129.67 ± 10.33	0.582
PLT (10^9^/L)	312.25 ± 107.62	275.12 ± 96.86	0.001
CRP (ug/ml)	2.93 (0.75 ~ 7.34)	7.50 (3.65 ~ 21.08)	< 0.001
ALT (U/L)	14.80 (11.40 ~ 18.90)	18.20 (14.15 ~ 29.00)	< 0.001
AST (U/L)	31.40 (26.30 ~ 39.80)	30.90 (23.60 ~ 42.50)	0.937
LDH (U/L)	301.00 (257.60 ~ 359.00)	311.90 (265.45 ~ 446.15)	0.030
CK (U/L)	90.05 (59.50 ~ 129.00)	80.00 (52.95 ~ 141.00)	0.424
CK-MB (U/L)	24.00 (16.40 ~ 36.10)	20.00 (12.05 ~ 35.10)	0.015
D-d (mg/L)	0.38 (0.25 ~ 0.57)	0.89 (0.50 ~ 1.75)	< 0.001
Chest imaging score	411	131	< 0.001
1	316(76.88%)	55(41.98%)	
2	71(17.27%)	25(19.08%)	
3	19(4.62%)	19(14.50%)	
4	5(1.22%)	32(24.43%)	

Normal distribution data was presented as mean ± standard deviation. Skewness distribution data was presented as medians (one-quarter ~ three-quarters). *P* < 0.05 was considered statistically significant

*Abbreviations*: *WBC* white blood cells, *PMNs* polymorphonuclear neutrophils, *L* lymphocytes, *Hb* hemoglobin, *PLT* platelet, *CRP* C-reactive protein, *AST* glutamic oxaloacetic transaminase, *ALT* glutamic-pyruvic transaminase, *LDH* L-lactate dehydrogenase, *CK* creatine kinase, *CK-MB* creatine kinase-MB, *D-d* D-dimer

**Table 4:** Admission characteristics of children with *Mycoplasma pneumoniae* pneumonia in the validation cohort

Items	Non-RMPP (*n* = 258)	RMPP (*n* = 64)	*P*-value
Age (years)	4.25 (2.17 ~ 7.00)	12.00 (8.00 ~ 12.00)	< 0.001
Fever days	3.00 (1.00 ~ 4.00)	7.00 (5.00 ~ 10.00)	< 0.001
WBC (10^9^/L)	8.39 (6.19 ~ 11.92)	8.90 (6.85 ~ 11.15)	0.447
PMNs %	56.17 ± 17.62	65.37 ± 14.03	< 0.001
L %	34.11 ± 16.20	25.78 ± 12.65	< 0.001
PMNs (10^9^/L)	4.49 (2.96 ~ 6.69)	5.51 (4.17 ~ 7.97)	0.007
L(10^9^/L)	2.42 (1.78 ~ 3.67)	2.17 (1.50 ~ 3.20)	0.025
Hb (g/L)	129.11 ± 9.91	129.67 ± 10.33	0.582
PLT (10^9^/L)	312.25 ± 107.62	275.12 ± 96.86	0.001
CRP (ug/ml)	3.56 (1.07 ~ 10.23)	16.45 (4.25 ~ 28.10)	< 0.001
ALT (U/L)	14.10 (10.90 ~ 17.80)	15.00 (11.50 ~ 23.50)	0.103
AST (U/L)	33.90 (26.60 ~ 40.70)	29.00 (23.20 ~ 38.50)	0.009
LDH (U/L)	315.40 (266.60 ~ 392.00)	393.00 (285.50 ~ 530.50)	0.002
CK (U/L)	91.00 (61.30 ~ 132.00)	72.00 (46.50 ~ 131.00)	0.049
CK-MB (U/L)	23.55 (10.80 ~ 37.00)	0.86 (0.67 ~ 1.51)	< 0.001
D-d (mg/L)	0.35 (0.22 ~ 0.53)	1.28 (0.90 ~ 3.96)	< 0.001
Chest imaging score	268	84	< 0.001
1	218(81.34%)	37(44.05%)	
2	41(15.30%)	22(26.83%)	
3	9(3.36%)	3 (3.57%)	
4	0(0.00%)	22(26.83%)	

Normal distribution data was presented as mean ± standard deviation. Skewness distribution data was presented as medians (one-quarter ~ three-quarters). *P* < 0.05 was considered statistically significant

*Abbreviations*: *WBC* white blood cells, *PMNs* polymorphonuclear neutrophils, *L* lymphocytes, *Hb* hemoglobin, *PLT* platelet, *CRP* C-reactive protein, *AST* glutamic oxaloacetic transaminase, *ALT* glutamic-pyruvic transaminase, *LDH* L-lactate dehydrogenase, *CK* creatine kinase, *CK-MB* creatine kinase-MB, *D-d* D-dimer

**Table 5:** Risk factor analysis of refractoy. *Mycoplasma pneumoniae* pneumonia in a modeling cohort

Items	Univariate analysis		Multivariates analysis	
	**HR(95% CI)**	***P*-value**	**HR(95% CI)**	***P*-value**
Age (years)	1.29 (1.18 ~ 1.41)	< 0.001	1.16 (1.08 ~ 1.33)	0.038
Fever days	1.33 (1.23 ~ 1.44)	< 0.001	1.34 (1.20 ~ 1.50)	< 0.001
WBC (10^9^/L)	0.94 (0.89 ~ 1.00)	0.038		
PMNs %	1.04 (1.03 ~ 1.06)	< 0.001		
L %	0.95 (0.93 ~ 0.97)	< 0.001		
PMNs (10^9^/L)	1.04 (0.98 ~ 1.11)	0.227		
L (10^9^/L)	0.52 (0.43 ~ 0.64)	< 0.001	0.45 (0.23 ~ 0.89)	0.021
Hb (g/L)	1.01 (0.99 ~ 1.03)	0.581		
PLT (10^9^/L)	1.00 (1.00 ~ 1.00)	0.001		
CRP (ug/ml)	1.04 (1.03 ~ 1.06)	< 0.001		
ALT (U/L)	1.03 (1.02 ~ 1.05)	< 0.001		
AST (U/L)	1.01 (1.00 ~ 1.02)	0.045		
LDH (U/L)	1.00 (1.00 ~ 1.00)	0.003		
CK (U/L)	1.00 (1.00 ~ 1.00)	0.091		
CK-MB (U/L)	0.99 (0.98 ~ 1.00)	0.069		
D-d (mg/L)	2.50 (1.88 ~ 3.33)	< 0.001	1.70 (1.16 ~ 2.49)	0.006
Chest imaging score	6.56 (3.52 ~ 12.24)	< 0.001	5.16 (2.38 ~ 11.21)	< 0.001

*P* < 0.05 was considered statistically significant

*Abbreviations*: *WBC* white blood cells, *PMNs* polymorphonuclear neutrophils, *L* lymphocytes, *Hb* hemoglobin, *PLT* platelet, *CRP* C-reactive protein, *AST* glutamic oxaloacetic transaminase, *ALT* glutamic-pyruvic transaminase, *LDH* L-lactate dehydrogenase, *CK* creatine kinase, *CK-MB* creatine kinase-MB, *D-d* D-dimer

### Development and validation of the predictive nomogram model for RMPP

**Fig. 3:**
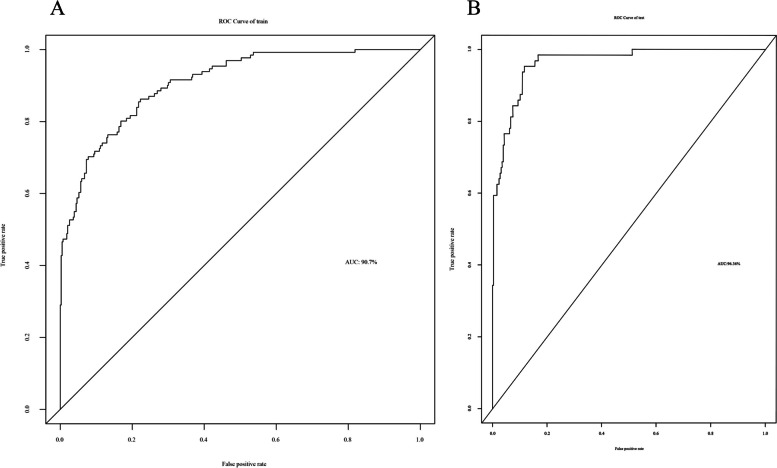
Receiver operating characteristic curves to predict refractory *Mycoplasma pneumoniae* pneumonia. **A** The modeling cohort and **B** the validation cohort

**Fig. 4:**
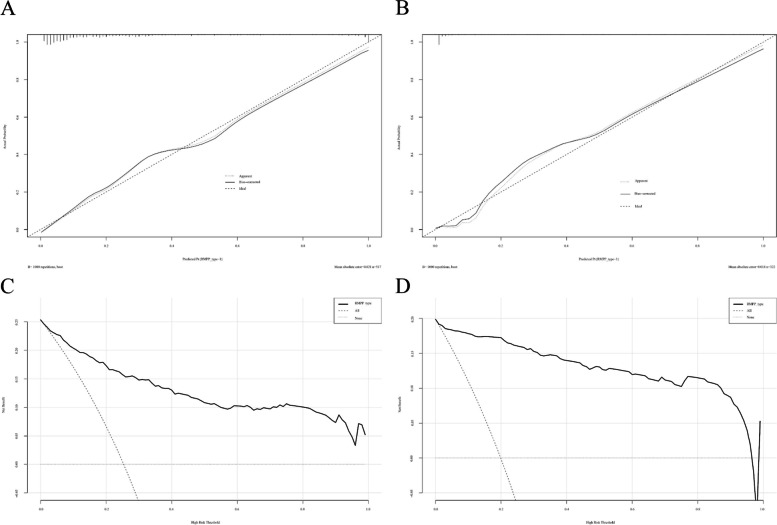
Calibration and validation curve to predict refractory *Myocplasma pneumoniae* pneumonia. The calibration curve in the modeling (**A**) and validation (**B**) cohorts. The nomogram model to predict RMPP is plotted on the x-axis, and the actual RMPP is plotted on the y-axis. The reference line is 45° and indicates perfect calibration. The validation curve in the modeling (**C**) and validation (**D**) cohorts. The y-axis indicates the net benefit. The straight line represents the assumption that all patients will develop RMPP, and the horizontal line represents the assumption that no patient will develop RMPP

Based on the above results of the multivariate regression analysis, the final predictive model for the prognostic risk of RMPP was established and shown as a nomogram (Fig. 2B). The sum of the corresponding scores for age, fever duration, lymphocyte count, D-dimer level, and radiological imaging change in this nomogram was associated with an increased risk of RMPP. As an example case in which the nomogram was applied, a 6-year-old patient with MPP had fever for 5 days at admission, a lymphocyte count of 5 × 10^9^/L, and a D-dimer level of 6 mg/dL. The patient’s chest X-ray findings showed massive consolidation in the middle and lower lobes of the right lung, as well as a small amount of right-sided pleural effusion. The predictive variables produced the following number of points in the nomogram model: age of 6 years: 6 points; 5-day fever: 10 points; lymphocyte count: 20 points; D-dimer level: 21 points; chest imaging score of 3 points: 30 points. Therefore, this patient had a total score of 87 points. According to the RMPP type corresponding to the total score at the bottom of the chart, the probability of RMPP was approximately 98.0%. The area under the ROC curve was 0.907 in the modeling cohort (Fig. 3A), and 0.964 in the validation cohort (Fig. 3B), which indicated a good discrimination ability of the nomogram. The calibration curve was almost a straight line, which showed that the prediction model had a good fit in the modeling cohort (Fig. 4A) and the validation dataset (Fig. 4B). The DCA curve showed an obvious net benefits with the predictive nomogram and proved that the prediction model had good accuracy (Fig. 4C, D).

## Discussion

RMPP is associated with numerous potentially long-lasting sequela, so early recognition and effective management are vital. The excessive host immune responses as well as macrolide resistance of *Mycoplasma pneumoniae* [4] might play the important roles in RMPP progression. However, deleterious inflammatory responses is difficult to accurately identify in patients with MPP. Therefore to establish a quantitative tool might contribute to anticipating RMPP risk among patients with MPP. In this study, a predictive nomogram combined five independent risk factors (older age, prolonged fever, higher serum D-dimer level, higher lymphocyte count, and more severe radiological manifestations) were identified and constructed.

Numerous literatures have reported the correlation between age and RMPP. Children over the age of 5 years are more susceptible to *Mycoplasma pneumoniae* and exhibit more severe MPP symptoms [6, 18]. In our modeling cohort, the incidence of RMPP increased with age, suggesting that RMPP-associated lung injury is due to an age-related abnormal inflammatory response. Persistent high fever is also thought to be associated with an excessive inflammatory reaction to *Mycoplasma pneumonia* infection and concomitant lung damage. Choi et al. reported a longer fever duration, greater pleural effusion, and more severe lobar pneumonia along with higher incidences of extrapulmonary manifestations and atelectasis among patients with macrolide-resistant MPP compared with patients with macrolide-sensitive MPP [19]. Consistent with these findings, fever duration and initial radiological findings were predictive indicators of RMPP among MPP, both in the current study and in a previous investigation [17].

The lymphocyte count decreases in MPP to combat *Mycoplasma pneumoniae* infection in peripheral blood. Specifically, CD3^+^, CD4^+^, and CD79^+^ lymphocytes are recruited to the lungs, followed by enhanced humoral immunity. In fact, lymphocyte activation may be essential for the elimination of *Mycoplasma pneumoniae* [20]. Yan et al. reported that a lymphocyte percentage of ≤ 32% was associated with a higher risk of corticosteroid-resistant RMPP, and that glucocorticoid pulse therapy was effective against prolonged high fever [21], which is consistent with our findings showing that lymphocyte was a protective factor against airway injury in RMPP.

The serum D-dimer level was previously found to be an indicator of the severity of community-acquired pneumonia [22]. Similarly, D-dimer levels have been shown to be higher in severe cases of MPP than in mild cases [10, 23]. Moreover, increased D-dimer level was more suspicious of *Mycoplasma pneumoniae* associated necrotizing pneumonia [24]. The present study revealed that D-dimer is a predictor of RMPP, which is consistent with previous reports [25]. This association indicates that vascular endothelial injury and abnormal coagulation mechanisms are involved in the pathogenesis of RMPP.

The performance of the nomogram incorporating D-dimer level and the other four clinical variables that were identified as independent risk factors was satisfactory with good discrimination and accuracy in both the development and validation cohorts as evidenced by the ROC curve analysis. Additionally, the nomogram demonstrated a high overall net benefit in both the modeling and validation cohorts. The five variables used to construct the nomogram are easily obtained at admission, this tool could help to early estimate RMPP risk in pediatric patients with MPP, and enable effective therapy to begin sooner, and potentially improve clinical outcomes of RMPP. If a case with macrolide-unresponsive MPP, the use of antibiotic prescription, doxycycline or minocycline [26], and/or high-dose systemic corticosteroid [27], may alleviate the clinical symptoms of RMPP, and prevent disease progression from MPP into RMPP. If the level of D-dimer is elevated, low molecular weight heparin can reduce the risk of pulmonary embolism in children with RMPP [24]. If RMPP cases were complicated with atelectasis, or large masses of solid malabsorption, the combined bronchoalveolar lavage intervention is a better treatment for those patients [28].

In contrast to our results, Cheng et al. identified LDH, albumin, PMNs ratio, and high fever as independent predictive factors for RMPP risk among pediatric patients with MPP [29]. Another study showed that age combined with fever days, CRP, GOT, LDH, and chest imaging score effectively identified RMPP early [17]. Compared with Cheng et al.’s RMPP predictive model, clinical variables were added to construct the model in the present study, and in contrast with Bi et al.’s model, an additional factor, D-dimer, was included to enhance the diagnostic accuracy of our model. Therefore, our nomogram showed more excellent discrimination.

The current study has some limitations, including the retrospective design, which may have introduced selection bias and precluded any analysis on causal relationships. Therefore, a prospective cohort study is required to verify the clinical utility of the proposed predictive nomogram. Moreover, some variables of possible clinical significance, such as extrapulmonary manifestations and macrolide unresponsiveness, were not included as variables in the RMPP nomogram model. Despite these limitations, this nomogram model demonstrated its effectiveness as a tool for the early identification of RMPP among patients with MPP based on five easily obtainable risk factors. In turn, early diagnosis could allow for the initiation of timely and effective therapy to improve RMPP outcomes.

## Conclusion

In this study, a nomogram that incorporated age, fever duration, lymphocyte count, D-dimer level, and chest imaging findings was constructed, which was able to accurately predict the progression from MPP to RMPP for early diagnosis and timely treatment. The established nomogram is a simple and easy-to-use scoring system for application in clinical practice.

### Supplementary Information

**Additional file 1.** TRIPOD checklist: prediction model development and validation.

**Additional file 2.** The Criteria of MPP.

**Additional file 3.** R Software usage.

**Additional file 4.** R Software Pmsampasize usage.

## Data Availability

The datasets generated and/or analysed during the current study are not publicly available within 2 years due to master dissertation. The corresponding author should be contacted to request the datasets on reasonable request.

## Abbreviations

RMPP: Refractory *M**ycoplasma** pneumoniae* pneumonia
Non-RMPP: Without refractory *M**ycoplasma** pneumoniae* pneumonia
MPP: *M**ycoplasma** pneumoniae* Pneumonia
WBC: White blood cells
PMNs: Polymorphonuclear neutrophils
PLT: Platelets
Hb: Hemoglobin
CRP: C-reactive protein
GOT: Glutamic-oxaloacetic transaminase
GPT: Glutamic-pyruvic transaminase
LDH: L-lactate dehydrogenase
CK: Creatine kinase
CK-MB: Creatine kinase-MB
ROC: Receiver operating characteristic
DCA: Decision curve analysis
D-d: D-dimer
