# Supplementary material for: Recognition of refractory Mycoplasma pneumoniae pneumonia among Myocoplasma pneumoniae pneumonia in hospitalized children: development and validation of a predictive nomogram model
Source: BMC Pulm Med. 2023 Oct 10;23:383. doi: 10.1186/s12890-023-02684-1 (PMC10566172; doi:10.1186/s12890-023-02684-1)
Supplement: Supplementary file 2 — Additional file 2. The Criteria of MPP. [file 12890_2023_2684_MOESM2_ESM.doc]

The Criteria of MPP

According to the Chinese Expert Consensus on Diagnosis and Treatment of MPP in Children (2015 Edition), the diagnosis of MPP was based on following: (a) clinical symptoms and signs, such as cough, fever, wheezing, dyspnea,chest discomfort, and abnormal lung auscultation. (b) imaging changes of pulmonary parenchymal and/or interstitial inflammatory infiltration. (c) positive laboratory results for *M. pneumoniae*, including an *M. pneumoniae* -IgM titer ≥ 1:160 or four-fold rising titer in acute and convalescent serum specimen; positive PCR result for *M. pneumoniae* in the fuid of BAL, or pleural efusion, or nasopharyngeal swab specimmen, was a copy number of > 3,000/mL (Ningbo Health Gene Technologies Co., Ltd., Ningbo, China). (d) the negative test results of other pathogens, such as nasopharyngeal aspirate and blood culture, enzyme-linked immunospot assay of *Mycobacterium tuberculosis*, and 12 respiratory pathogens of nasopharyngeal aspirate using reverse transcription PCR examinations, including *influenza virus A, influenza virus A (H1N1), seasonal influenza virus (H3N2), influenza virus B, respiratory syncytial virus, parainfluenza virus, metapneumovirus, adenovirus, rhinovirus, bocavirus,* *chlamydia,* and *coronavirus*.
