# Supplementary material for: Recognition of refractory Mycoplasma pneumoniae pneumonia among Myocoplasma pneumoniae pneumonia in hospitalized children: development and validation of a predictive nomogram model
Source: BMC Pulm Med. 2023 Oct 10;23:383. doi: 10.1186/s12890-023-02684-1 (PMC10566172; doi:10.1186/s12890-023-02684-1)
Supplement: Supplementary file 3 — Additional file 3. R Software usage. [file 12890_2023_2684_MOESM3_ESM.doc]

**R Software usage**

``{r setup, include = FALSE}

knitr::opts_chunk$set (echo = F,warning = F,message = F)

Library (foreign)

Library (rms)

Library (readxl)

Library (rmda)

Library (Resource Selection)

Library (ROCR)

Library (forest plot)

Library (forest model)

```

1. Reading file

```{r,warning=FALSE,message=FALSE}

mydata<-read_excel("modeling cohort data .xlsx")

mydata<-data.frame(mydata)

## The first 6 lines is showed.

head(mydata)

```

## 2.Extract modeling data

```{r}

mydata1<-mydata [,c (1,3,4,9,18,19)]

## rename

Names (mydata1) <-c ("RMPP_type","age","Fever_time", "L","D_d", "RA_score")

Head (my data1)

```

## 3. logistic regression

The OR value is the index of the regression coefficient. For example, the OR of age of exp (0.1419) is equal to 1.152461

```{r}

Attach (my data1)

## To load the data frame into the current working environment

Datadist could pack data

dd<-datadist (mydata1)

Options (datadist='dd')

fit1<-lrm (`RMPP_type` ~ ., data = mydata1, x = T, y = T)

fit1

Detach (my data1)

```

## 4. Nomogram

```{r,warning=FALSE,message=FALSE}

nom1 <- nomogram(fit1, fun = plogis,fun.at = c (.001, .01, .05,

Seq (.1,.9, by = .2), .95, .99, .999),

lp = F, funlabel = "RMPP_type")

Plot (nom1)

```

## Forrestplot

```{r}

mod<-glm (RMPP_type~age+Fever_time+L+D_d+RA_score,data=mydata1,family = binomial (link = "logit"))

forest_model (mod)

```

## 5. The calibration curve of internal validation

```{r,warning=FALSE,message=FALSE}

cal1 <- calibrate (fit1, method = 'boot', B = 1000)

Plot (cal1,xlim = c (0,1.0),ylim = c (0,1.0))

```

## 6. Decision curve of the modeling group

```{r}

simple<-decision_curve (RMPP_type~age+Fever_time+L+D_d+RA_score,data=mydata1,family = binomial (link = "logit"))

plot_decision_curve (simple,confidence.intervals = F,standardize = F,curve.names = "RMPP_type",cost.benefit.axis = F,col = c ("black","black","black"), lty = c (1,2,3))

```

## 7. ROC curve of the modeling group

```{r}

pr<-predict (mod,mydata1,type = c ("response"))

ROCR predlg <- ROCR:: prediction (pr, mydata1$RMPP_type)

ROCR pelglg <- performance (ROCRpredlg, "tpr", "fpr")

ROCR auclg <- performance (ROCRpredlg, measure = "auc")

Par (mar = c(5, 3, 2,3),pty='s')

Plot (ROCRpelglg, colorize=F,

text.adj= c (-0.2, 1.7),main = "ROC Curve of train")

Abline (a = 0,b = 1)

Text (x=0.8,y=0.4, paste ("AUC: ", 100*round (as.numeric (ROCRauclg@y.values),

digits = 4),

"%", sep = ""), col = "black")

```

## 8. Fetch from the test set

```{r}

Test <-read_excel ("validation group.xlsx")

test1 <-test [,c(1,3,4,9,18,19)]

## Rename

Names (test1) < -c ("RMPP_type","age","Fever_time", "L","D_d", "RA_score")

Head (test1)

```

## 9. Decision curve of the validation group

```{r}

Simple < -decision_curve (RMPP_type~age + Fever_time+L+D_d+RA_score, data = test1, family = binomial (link = "logit"))

plot_decision_curve (simple,confidence.intervals = F, standardize = F, curve.names = "RMPP_type", cost. benefit. axis = F, col = c ("black","black","black"), lty = c(1,2,3))

```

## 10. ROC curve of the validation data

```{r}

Pr < -predict (mod,test1,type = c("response"))

ROCRpredlg <- ROCR::prediction (pr, test1 $RMPP_type)

ROCRpelglg <- performance (ROCRpredlg, "tpr", "fpr")

ROCRauclg <- performance (ROCRpredlg, measure = "auc")

Par (mar = c(5, 3, 2,3), pty='s')

Plot (ROCRpelglg, colorize = F,

text.adj = c(-0.2, 1.7), main = "ROC Curve of test")

Abline (a = 0,b = 1)

Text (x=0.8,y=0.4, paste ("AUC: ", 100 * round (as.numeric (ROCRauclg@y.values), digits = 4),

"%",sep = ""), col = "black")
