# Supplementary material for: Recognition of refractory Mycoplasma pneumoniae pneumonia among Myocoplasma pneumoniae pneumonia in hospitalized children: development and validation of a predictive nomogram model
Source: BMC Pulm Med. 2023 Oct 10;23:383. doi: 10.1186/s12890-023-02684-1 (PMC10566172; doi:10.1186/s12890-023-02684-1)
Supplement: Supplementary file 4 — Additional file 4. R Software Pmsampasize usage. [file 12890_2023_2684_MOESM4_ESM.docx]

**R Software Pmsampasize usage**

> pmsampasize(

+ type=“b”

+ rsquared=0.47

+ parameters=5

+ prevalence=0.1

+ seed=123456)

NB= Assuming 0.05 acceptable difference in apparent ＆ adjusted R-squared

NB= Assuming 0.05 margin of error in estimation of intercept

NB= Events per Predictor Parameter (EPP) assumes prevalence= 0.098

Samp＿size Shrinkage Parameter CS＿Req Max＿Req Neg＿Req EPP

Criteria 1 68 0.900 5 0.47 0.473 0.933 1.33

Criteria 2 152 0.952 5 0.47 0.473 0.933 3.02

Criteria 3 136 0.952 5 0.47 0.473 0.933 2.67

Final 152 0.952 5 0.47 0.473 0.993 3.02

Minimum sample size required for new model development based on user inputs with 16 events

(assuming an outcome prevalence = 0.1) and an EPP =3.02

pmsampasize(

+ type=“b”

+ rsquared=0.7

+ parameters=5

+ prevalence=0.3

+ seed=123456)

NB= Assuming 0.05 acceptable difference in apparent ＆ adjusted R-squared

NB= Assuming 0.05 margin of error in estimation of intercept

NB= Events per Predictor Parameter (EPP) assumes prevalence= 0.3

Samp＿size Shrinkage Parameter CS＿Req Max＿Req Neg＿Req EPP

Criteria 1 34 0.900 5 0.7 0.705 0.933 2.04

Criteria 2 79 0.952 5 0.7 0.705 0.933 4.74

Criteria 3 323 0.952 5 0.7 0.705 0.933 19.38

Final 323 0.952 5 0.7 0.705 0.993 19.38

Minimum sample size required for new model development based on user inputs with 97 events

(assuming an outcome prevalence = 0.3) and an EPP =19.3
